# A Novel *Botrytis* Species Is Associated with a Newly Emergent Foliar Disease in Cultivated *Hemerocallis*

**DOI:** 10.1371/journal.pone.0089272

**Published:** 2014-06-02

**Authors:** Robert T. Grant-Downton, Razak B. Terhem, Maxim V. Kapralov, Saher Mehdi, M. Josefina Rodriguez-Enriquez, Sarah J. Gurr, Jan A. L. van Kan, Frances M. Dewey

**Affiliations:** 1 Department of Plant Sciences, University of Oxford, Oxford, England; 2 Wageningen University, Laboratory of Phytopathology, Wageningen, The Netherlands; 3 Instituto de Bioorgánica Antonio González (IUBO) University of La Laguna, Tenerife, Spain; Institute for Plant Protection (IPP), CNR, Italy

## Abstract

Foliar tissue samples of cultivated daylilies (*Hemerocallis* hybrids) showing the symptoms of a newly emergent foliar disease known as ‘spring sickness’ were investigated for associated fungi. The cause(s) of this disease remain obscure. We isolated repeatedly a fungal species which proved to be member of the genus *Botrytis*, based on immunological tests. DNA sequence analysis of these isolates, using several different phyogenetically informative genes, indicated that they represent a new *Botrytis* species, most closely related to *B. elliptica* (lily blight, fire blight) which is a major pathogen of cultivated Lilium. The distinction of the isolates was confirmed by morphological analysis of asexual sporulating cultures. Pathogenicity tests on *Hemerocallis* tissues *in vitro* demonstrated that this new species was able to induce lesions and rapid tissue necrosis. Based on this data, we infer that this new species, described here as *B. deweyae,* is likely to be an important contributor to the development of ‘spring sickness’ symptoms. Pathogenesis may be promoted by developmental and environmental factors that favour assault by this necrotrophic pathogen. The emergence of this disease is suggested to have been triggered by breeding-related changes in cultivated hybrids, particularly the erosion of genetic diversity. Our investigation confirms that emergent plant diseases are important and deserve close monitoring, especially in intensively in-bred plants.

## Introduction

The emergence of new fungal pathogens in cultivated and wild plants is a major cause for concern [1] and many disparate factors influence such patterns of emergence and establishment. Interspecific hybridisation between different fungal species may be a major contributor to the evolution of novel diversity [2]. In plant pathogens, it has been demonstrated recently that unusually rapid genome evolution and host shifts may occur after hybridisation events. For example, establishment of a new species of *Zymoseptoria, Z. pseudotritici,* exhibiting an expanded host range on grasses took place within just a few hundred generations of a hybridisation event between *Z. tritici* and an unidentified species [3]. There is also considerable evidence that the commensal endophytic state and a parasitic, pathogenic state can be highly plastic [2], [4]. Indeed, there is confirmation that several fungal diseases of plants can exist as a symptomless, endophytic infection – as, for instance, in the necrotrophic generalist *Botrytis cinerea* (grey mould) [5],[6] and obligate biotrophic *Albugo* species [7]. Anthropogenic change to natural environments is also likely to be a major factor in promoting the emergence of new pathogens [1]. Important issues such as climate change, degradation of natural environments (such as forest clearance and loss of genetic diversity in natural plant populations), dispersal of plants and their associated fungi to new areas and dependence on agricultural monoculture systems are likely to dramatically enhance the probability of new diseases emerging, establishing and spreading. The initial emergence events are likely to go unreported and uninvestigated not only because of their infrequency, but also because the damage resulting from a new pathogen could be casually attributed to other microbial species or to abiotic stress-induced damage.

The genus *Hemerocallis* (daylily) of the family Hemerocallidaceae in the order Asparagales has been cultivated for thousands of years [8]. This small genus of petaloid monocotyledons has been valued extensively as a food crop, as a medicinal plant and as an ornamental. The native range extends from eastern Asia, with a centre of diversity in Japan, Korea and China, possibly as far west as central Europe. Widespread cultivation has led to naturalisation of the genus in many parts of the world [8]. Cultivated *Hemerocallis* in North America, where the numerous hybrids are exceptionally popular ornamental garden plants, were recently challenged by the accidental introduction of a genus-specific biotrophic rust pathogen, *Puccinia hemerocallidis* (daylily rust) [9], [10]. This pathogen caused a rapidly-spreading epidemic. The lack of resistance amongst hybrid cultivars - with the one study showing only a minority of the cultivars tested exhibiting any resistance to even a single *P. hemerocallidis* isolate - demonstrated the susceptibility of cultivated varieties that have been bred outside of their native range in the absence of any pressure from this specific pathogen [11]. Other emerging threats to cultivated daylily include the first reports of infection by the *Armillaria* genus [12], [13]. Both in the West and in China, anthracnose diseases caused by various *Colletotrichum* species have been identified and a new species of *Colletotrichum, C. hemerocallidis*, was recently described from infected leaf and scape material in China [14].

However, the aetiology of one emerging disease of cultivated *Hemerocallis* in Western gardens has remained elusive. ‘Spring sickness’ describes a group of disease symptoms that primarily affect the emergent post-winter foliar growth of the plant, causing distortion, stunting, chlorosis, ragged leaf edges and necrotic lesions [15]. These symptoms not only disfigure the foliage but also weaken the plant, resulting in reducing flowering, and -in severe cases - the affected growing point dies. The first reports of this disease come from the U.S.A. in the 1970s [16] and widespread notification of these symptoms has occurred only over the past 20 years. To date, there has not been a report in peer-reviewed published literature of filamentous fungal species being isolated from *Hemerocallis* material showing such symptoms. Nevertheless, there is considerable speculation that fungal pathogens may be responsible or contribute to disease development [15], [16], [17].

Here, we report the isolation of a new species of *Botrytis* from foliar material of *Hemerocallis* from the United Kingdom that showed a range of symptoms that correspond to ‘spring sickness’. We demonstrate that this new species is distinct at the molecular and morphological level from its closest relative, *B. elliptica* (lily blight, fire blight), and that the two species have diverged in their host range. Our data shows that cryptic fungal species and perplexing fungal diseases can still be identified from common cultivated plants outside of their natural range, suggesting that there may be a significant reservoir of fungal diversity from which new diseases may emerge.

## Results

### Isolations and Preliminary Identification

**Figure 1:**
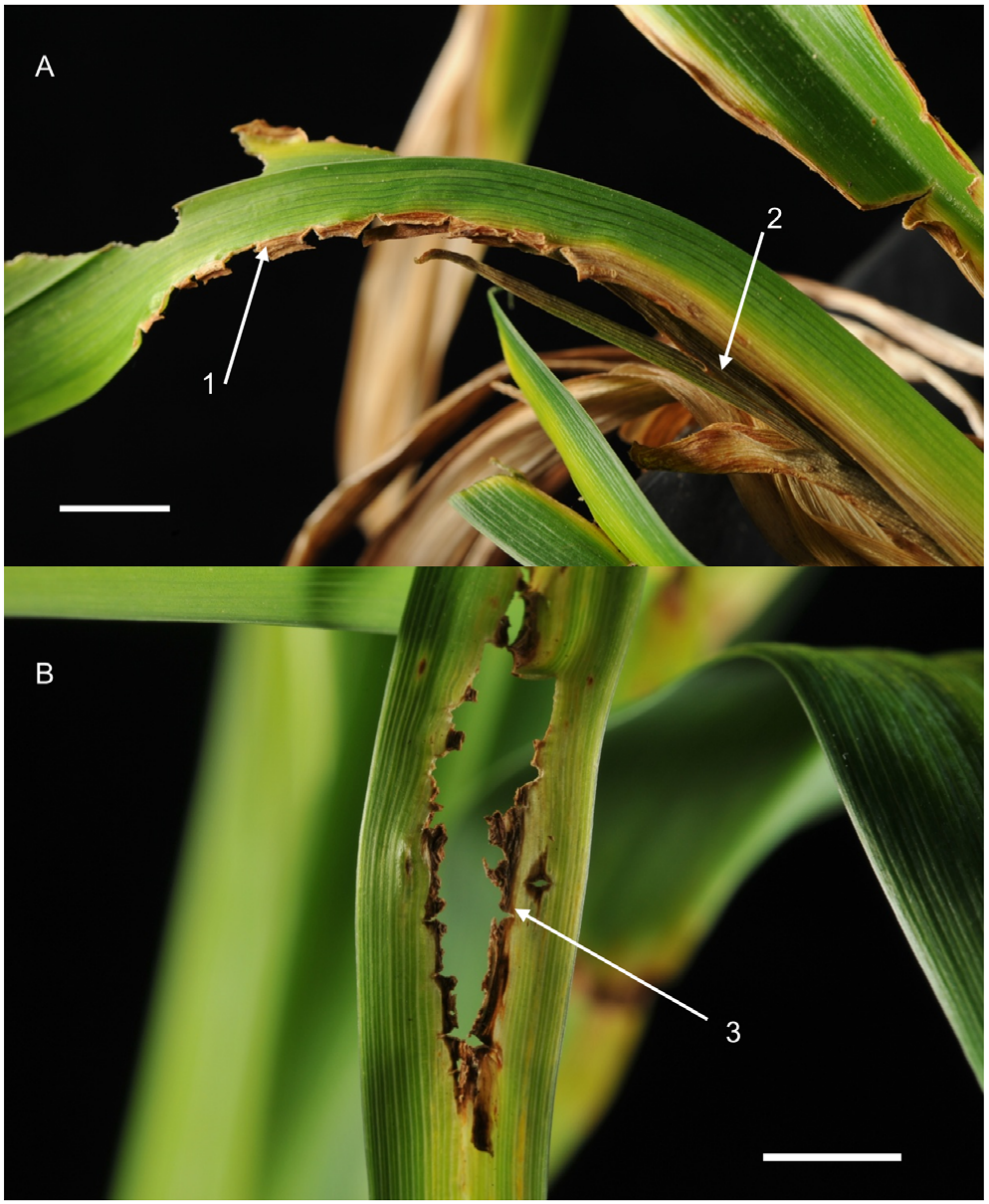
Spring foliage of *Hemerocallis* cultivars showing symptoms typical of ‘spring sickness’ disease. Areas of chlorosis, necrotic lesions and distortion of leaf development are typical of this disease. *Botrytis deweyae* was isolated from this material. Scale bars indicate 1 cm. A. Foliage of *Hemerocallis* ‘Free Bird’ showing advanced tissue necrosis with destruction of tissue at leaf margins (1) and death in the youngest emerging leaves (2). B. Leaf material of *Hemerocallis* ‘Lola Branham’ showing large necrotic lesions developing along the central parts of the leaf (3).

**Figure 2:**
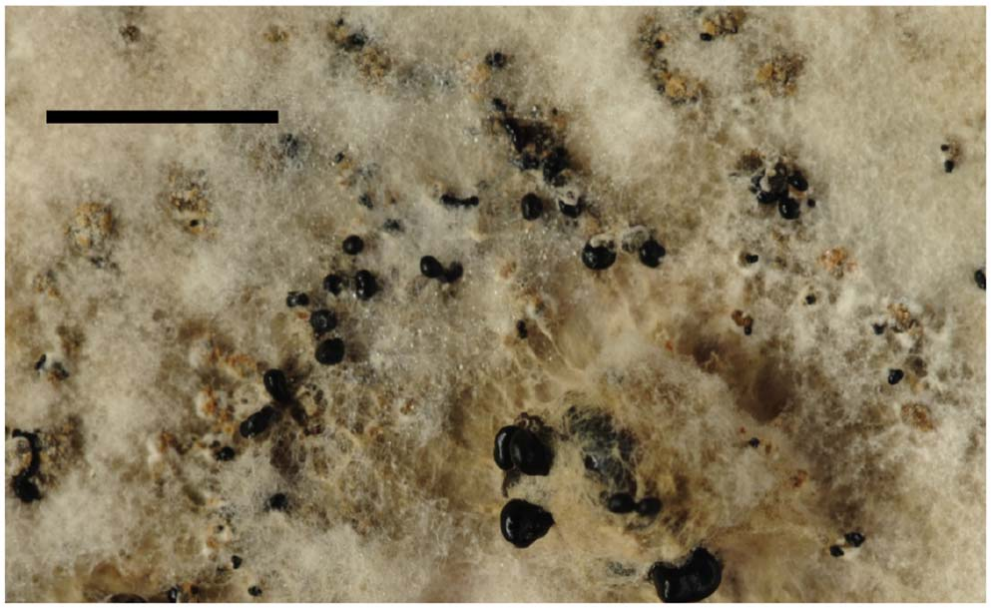
Development after isolation of *Botrytis deweyae* cultures from surface-sterilised *Hemerocallis* material. The initial development of multiple melanised sclerotia distributed across the colony surface can be seen in this maturing culture. Scale bar indicates 1

Over a period of 4 years, leaf samples of *Hemerocallis* cultivars that showed symptoms typical of or closely related to ‘spring sickness’ such as necrotic patches, necrotic lesions, distortion and chlorosis (Fig. 1a,b) were found to be associated with an unidentified filamentous fungus after the material was rigorously surface-sterilised and plated on malt extract agar (MEA). Occasionally, fungal growth was visible on the symptomatic plant material in the form of dense, short mycelia producing microconidia (Figure S1a,b) suggesting that a filamentous fungus triggers symptom development. However, no macroconidia could be observed on the plant material. In total, 6 independent isolates were collected (Table 1). Vegetative growth at room temperature (25°C) with ambient light and supplemental near-UV light was rapid, but significant macroconidial sporulation did not occur (Figure 2). Although microconidia were readily identified, only a very small number of macroconidia could be found on older cultures at the edges of the Petri dish in two isolates (B2 and B4). The overall morphology suggests the isolates are members of the genus *Botrytis.* Immunological tests of surface washings of plate cultures of the isolates using the *Botrytis*-specific monoclonal antibody BC-12.CA4 [18], [19], [20] gave strong positive results (Table 1) indicating that they are all members of the genus *Botrytis*. These results together with the appearance of *Botrytis*-like melanised sclerotia in older cultures (Figure 2) prompted further investigations and DNA analyses.

**Table 1:** Table of fungal isolates identified in this study.

Isolate name	Host name	Host symptoms	Date of isolation	Culture S.I.
B1	*Hemerocallis* ‘Jurassic Spider’	Developing necrosis, distortion and chlorosis of young foliage	30 November 2009	42
B2	*Hemerocallis* ‘Lola Branham’	Severe necrosis, distortion and chlorosis of young and maturing foliage	19 February 2010	35
B4	*Hemerocallis* unnamed tetraploid hybrid	Necrosis of mature outer leaves just above soil level	9 June 2010	11
B5	*Hemerocallis* ‘Gerda Brooker’	Severe necrosis of young and mature foliage	15 April 2011	50
P1	*Hemerocallis* ‘Free Bird’	Severe necrosis, distortion and chlorosis of aerial parts; eventual death of tissues back to rhizome	15 April 2011	55
B6	*Hemerocallis* ‘Ruby Storm’	Severe necrosis and chlorosis of leaves emerging from dormancy	12 March 2012	53

Host name, host symptoms and the date of isolation are described. The post-isolation fungal culture signal intensity (S.I.), from tests of PBST suface washings of fungal cultures, with EnviroLogix Botrytis QuickStix using the anti-*Botrytis*-monoclonal antibody BC-12.CA4, is shown.

### Sequence Analyses

**Figure 3:**
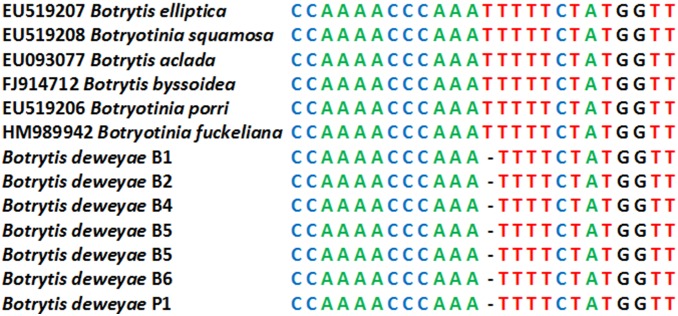
Alignment of a region of partial *ITS* sequence from various *Botrytis/Botryotinia* species. The aligned region shows the indel identified in all 6 examined isolates of *B. deweyae*.

To determine their identity, all 6 isolates were subjected to DNA sequence analysis. Sequencing of the *ITS* region confirm they all represent *Botrytis* species. Interestingly, all 6 isolates shared a unique polymorphism, an indel, in the highly conserved *ITS* sequence (Figure 3). BLAST analysis of Genbank data showed that no other known member of the genus possesses this sequence variant.

*ITS* sequence does not permit sufficient resolution to the species level. To further resolve the relationships of these isolates to other species in the genus *Botrytis*, analysis of nuclear protein-coding gene sequences that are known to be phylogenetically informative to the species level was undertaken. Five genes previously used to build phylogenies of the genus *Botrytis* [21], [22] were used. Three were single-copy housekeeping genes - encoding glyceraldehyde-3-phosphate dehydrogenase (*G3PDH*), a heat-shock protein (*HSP60*) and a DNA-dependent RNA polymerase subunit II protein (*RPB2*) [21] - and two were single-copy genes encoding proteins with roles in phytotoxicity (*NEP1* and *NEP2*) [22].

**Figure 4:**
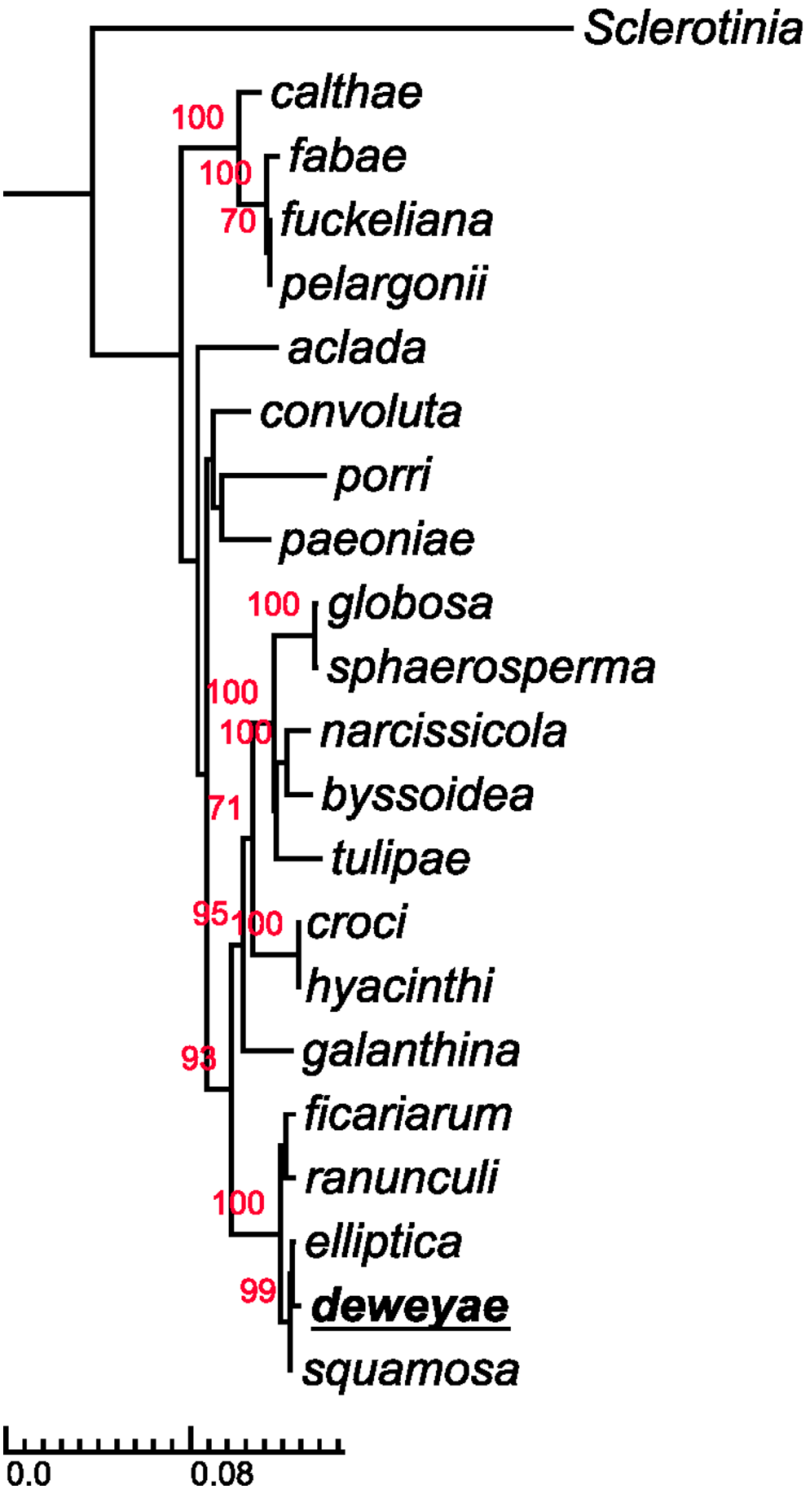
Phylogeny of *Botrytis* based on the combined analysis of 5 different genes. Sequences of *G3PDH*, *HSP60*, *RPB2*, *NEP1* and *NEP2* were used. The phylogenetic position of *B. deweyae* (B1 isolate, type) is underlined. The phylogeny was constructed using the genus *Sclerotinia* as the outgroup.

From the first isolate, B1, combined analysis of 5 sequences (*G3PDH*, *HSP60*, *RPB2*, *NEP1* and *NEP2*) confirmed the distinction (Figure 4). Analysis showed that except for HSP60 gene, all gene sequences showed sequence differences between B1 and other known members of *Botrytis* (Figures S2, S3, S4, S5, S6). The *HSP60* sequence was found to be identical to B. elliptica and the other sequences were also close to this species, suggesting that its nearest relation was *B*. *elliptica*. Phylogenetic analysis confirmed that this isolate was most closely related to *B*. *elliptica* and formed a monophyletic group with *B. elliptica* and *B. squamosa* (Figure 4). Divergence at synonymous sites between the new isolate and *B. elliptica* and *B. squamosa* was 1.1±0.32%, while divergence at synonymous sites between *B. elliptica* and *B. squamosa* was 1.0±0.31%.

For the other 5 isolates, two phylogenetically informative sequences (*G3PDH* and *NEP1*) were obtained. The presence of a polymorphism in the ITS1 sequence (Figure 3), as well as multiple sequence differences at other genes, strongly suggests that they represent an undescribed species [23]. *G3PDH*, *NEP1* and *ITS* sequences from all six isolates were identical confirming genetic similarity of isolates. Genetic similarity of isolates together with significant divergence between the new isolates and *B. elliptica* and *B. squamosa* strongly suggests that the new isolates represent an undescribed species.

Further supporting our notion that this species may be involved in development of ‘spring sickness’ symptoms, PCR amplification of a *NEP1* fragment from DNA extracts of symptomatic leaf material generated sequences corresponding to this natural phylogenetic group (Figure S7). Asymptomatic leaf material did not, however.

Sequences from fungal specimens were submitted to the Genbank database with accession numbers HG799518-HG799538.

### Morphological Analyses

Non-sporulating colonies are smooth to slightly fluffy and occasionally form aerial mycelia; colonies typically whitish to pale brown in colour on malt extract agar. The distance between septa ranges from 38.5 µm to 127 µm in length. Typically, sclerotia development is seen only as cultures mature; formation varies between isolates and depending on environmental conditions (Table S1 in File S1). Growth in darkness promotes sclerotia formation. On oatmeal agar, sclerotia are formed within 4 weeks at 15°C in darkness. Sclerotia are hemispherical convex in shape and with a concave surface, sometimes hollow in the centre. Sclerotia are black with size ranging from 2 to 6 mm (and on average 3 mm) in diameter.

**Figure 5:**
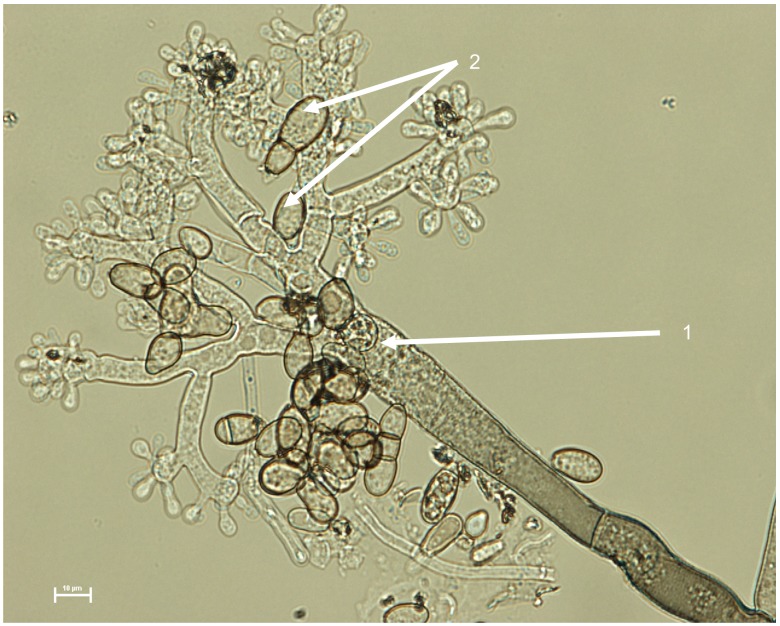
Development of *Botrytis deweyae* showing production of conidiophores and macroconidia. The conidiophore (1) terminating in a botryose cluster producing multiple macroconidia (2) is shown. Scale bar indicates 10 microns.

The initial absence of significant macroconidial development upon isolation indicates that such conditions are sub-optimal for promotion of asexual sporulation. Exclusion of light and exposure to supplemental near-UV light in the presence of daylight individually fails to trigger sporulation. Surface-sterilised host leaf material and sterile, crude extracts of host leaf material also do not stimulate sporulation. Sporulation is more reliably initiated after a minimum of 7 days exposure to near-UV light in the absence of other light sources, though this varies with the isolate and the medium (Table S2 in File S1). One isolate, B2, does not appear to sporulate under these conditions. The dependence on specific environmental conditions is a contrast to *B. elliptica* and *B. cinerea* which efficiently initiate macroconidia formation even when plates are exposed to daylight. The optimum temperature for sporulation of these isolates is 20°C. Conidiophores are erect, septate, opposite branched and slightly swollen at the top. The macroconidia develop in a botryose cluster and are oblong to spherical in shape (but sometimes ovoid), tapering and pointed at one end with their length in a range of 6.5 to 18 µm (mean 12.5 µm, *n* = 50) and width in a range of 3.5 to 11 µm (mean 7 µm, *n* = 50). Fungal material from cultures producing macroconidia are illustrated in Figure 5a,b. A scanning electron micrograph of the typical macroconidia is shown in Figure S8. A comparison to other described species can be found in Table S3 in File S1.

### Pathogenicity Assays

To determine whether *Botrytis* isolates induce the symptoms of the plant material from which they were originally isolated, basic assays were performed using excised surface-sterilised leaf material of *Hemerocallis* on plates. Inoculation of mycelial plugs directly onto the leaf surfaces resulted in a rapid formation of a spreading water-soaked lesion (Table S4 in File S1). Inoculation with *B. elliptica* did not produce visible lesions after the same period. Testing the novel isolates from *Hemerocallis* on the leaves of several other petaloid monocots such as *Lilium* did not produce lesions, indicating that the host range may be restricted. However, *B. elliptica* did induce similar lesions on *Lilium* material but not on *Hemerocallis* or any other species tested (Table S4 in File S1). Inoculation of *Hemerocallis* leaf material with conidial suspensions did not result in necrosis, though the conidia germinated and grew out into hyphae which after 10 days had covered the leaf surface.

**Figure 6:**
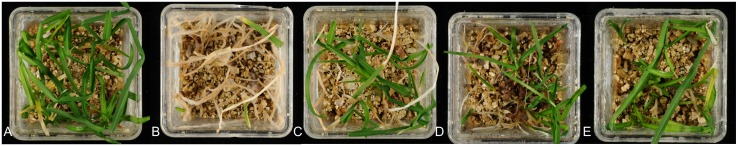
Sterile plantlets of *Hemerocallis* ‘Jurassic Spider’ inoculated with *Botrytis deweyae* and *Botrytis elliptica* isolates. Plantlets were grown in vermiculite with growth medium and are shown from above, 10 days after inoculations. A. *B. deweyae* B1 isolate. B. *B. deweyae* B2 isolate. C. *B. deweyae* B4 isolate. D. *B. elliptica*. E. control (no fungal inoculation).

**Figure 7:**
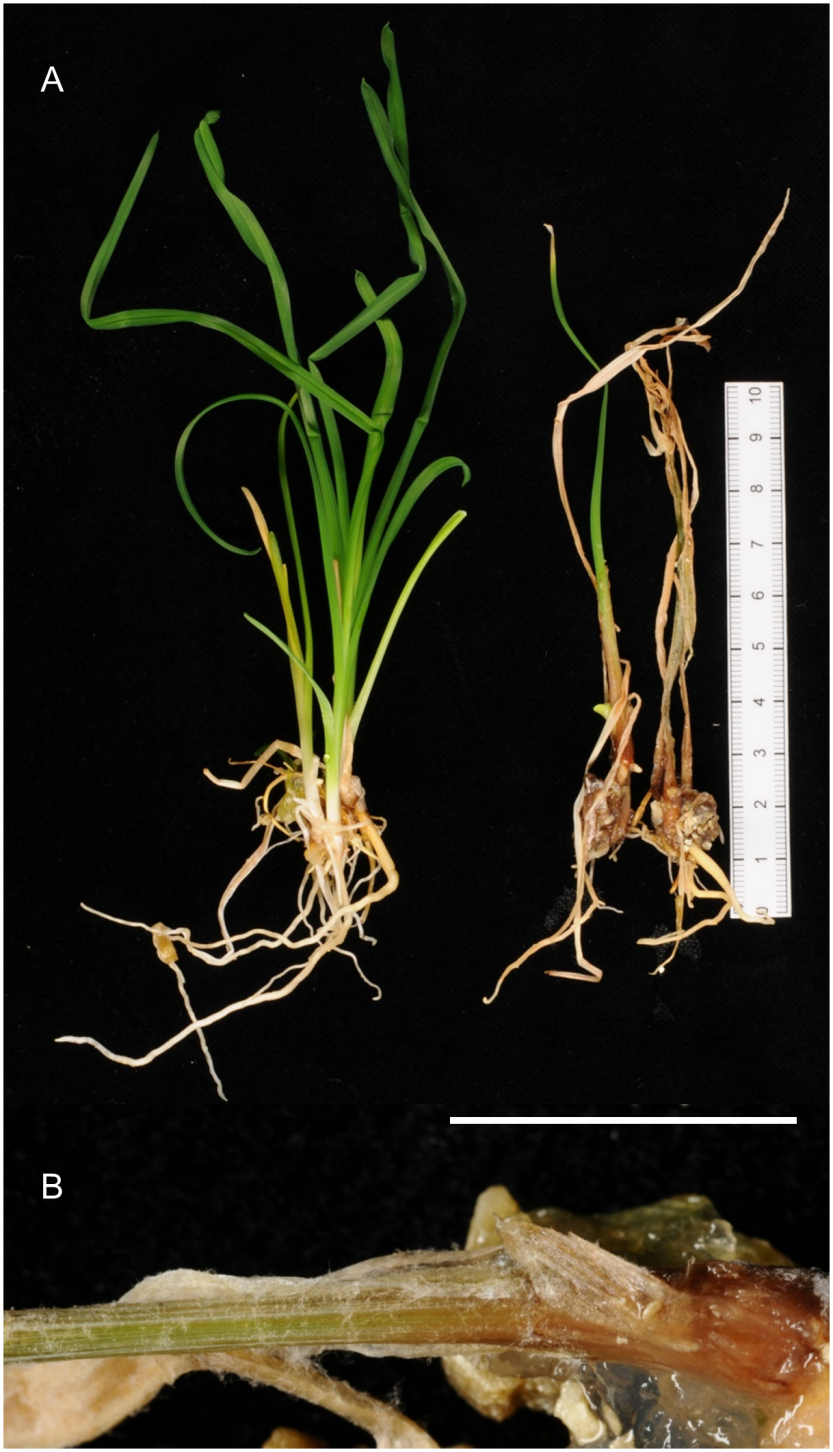
Effect of inoculation of *Hemerocallis* plantlets with *Botrytis deweyae*. A.Control *Hemerocallis* ‘Jurassic Spider’ plantlets (uninoculated with *Botrytis*) (left) and *Hemerocallis* ‘Jurassic Spider’ plantlets infected with *B. deweyae* (right) after inoculation with B1 isolate, shown 10 days after inoculation. The plant material was excised from sterile vermiculite. Scale indicates 10 cm. B. Close-up of tissue necrosis on the basal portion of a *Hemerocallis* ‘Jurassic Spider’ plantlet following *B. deweyae* infection, following colonisation of leaf bases. Scale indicates 5 mm.

Inoculation of *Hemerocallis* plantlets from *in vitro* culture demonstrated that these isolates had the capacity to cause severe necrosis and death of plantlets. Within 10*–*14 days of inoculation, plantlets showed chlorosis, necrosis, collapse and death of leaf tissue, although the roots and meristematic region were comparatively intact (Figure 6, Figure 7). Although these experiments on plantlets cannot exactly replicate the ‘spring sickness’ seen on mature plants, nevertheless the disease phenotypes are sufficient to satisfy basic Koch’s postulates. Tests on plant material extracts after inoculation with B1, B2 and B4 isolates using the *Botrytis*-specific monoclonal antibody BC-12.CA4 [18], [19], [20] gave strong positive results with signal intensity values ranging between 34 and 46 whilst the control plant tissue without fungal inoculation gave a signal intensity value of 0. B1 isolates appeared to be the most virulent, rapidly causing complete necrosis and collapse of all inoculated plant material within 14 days (Figure 6, Figure 7). Although microconidia could be found developing on leaf tissue, typical conidiophores producing macroconidia are not produced on the host under these conditions. Plantlets representing a range of different *Hemerocallis* cultivars were tested to determine if there may be easily recognised resistance to infection with isolate B1. All the tested cultivars developed similar symptoms of tissue necrosis, collapse and death within 14 days of inoculation.

### Mating Type

PCR analysis of the mating locus, *MAT1*, of *Botrytis* revealed that different isolates had different alleles (Table S5 in File S1). Reciprocal crosses between pairs of strains of opposite mating types, using a method well established for *B. cinerea* [24], [25], in all cases failed to result in the development of apothecia. Possibly this was due to the developmental condition of the sclerotia that had developed on plates and were used in the attempted sexual crosses in this study.

### Formal Description

Based on the molecular, morphological and host specificity data presented herein, this fungal isolate from *Hemerocallis* represents a new species:

#### Description

***Botrytis deweyae*** *van Kan, Terhem & Grant-Downton, sp. nov. MycoBank MB804656; Myconame ID 512235.*

*Conidiophores* erect, septate, medium brown, smooth, mostly unbranched, with slightly swollen basal and apical cells, 3–4 µm×10–20 µm. *Conidiogenous cells* predominantly terminal, pale brown, giving rise to botryose clusters of conidia. *Conidia* ellipsoid to ovoid, becoming oblong and 1-septate with age, or irregular and somewhat distorted, hyaline to medium brown, smooth, apex obtuse, base with small flattened abscission scar, 6.5–18 µm×3.5–11 µm (*av.*  = 12.5×7 µm, n = 50). No sporulation observed on leaf tissue. *Sclerotia* hemispherical, convex, sometimes hollow in the center, with concave surface; black, 2–6 mm (av. 3 mm) diam. Sclerotia develop in oatmeal agar within four weeks of incubation at 15°C in the dark. Sclerotia are scattered; cultures normally develop 25–30 sclerotia per plate.

*Culture characteristics*: Colonies incubated in the dark on MEA, optimal growth at 25°C, minimum at 4°C, maximum at 37°C. Colony growth rate 0.4–15.8 mm/d; surface dirty white to pale brown, reverse ochreous [26], with moderate to abundant aerial mycelium, creating fluffy appearance. Colonies also sporulate on MEA under near-ultraviolet light at 20°C.

*Typus*: UK, Oxford, from host plant *Hemerocallis* ‘Jurassic Spider’, Robert Grant-Downton, 30^th^ November 2009, holotype CBS H-21133; culture ex-type CBS 134650.

#### Distribution and host range

Known only from cultivated specimens of *Hemerocallis* from the UK but likely to be widespread on *Hemerocallis* in cultivation and possibly in the wild due to the cryptic nature of this species and its broad temperature tolerance.

#### Etymology

Named after Dr. Molly Dewey in recognition of her outstanding contributions to plant pathology and mycology, in particular relating to the genus *Botrytis*.

## Discussion

We report the discovery of a new species of the ascomycete fungal genus *Botrytis* from cultivated material of *Hemerocallis* (commonly known as the daylily) showing a range of symptoms corresponding to those reported for a mysterious disease, ‘spring sickness’. Recently, several new species of *Botrytis* have been reported, such as *B. sinoallii* from China which is pathogenic on *Allium* crops [27], *B. fabiopsis* from central China which is pathogenic on broad bean [28] and *B. caroliniana* from North America which is pathogenic on blackberry fruits and broad bean leaves [29]. Other as yet undescribed pathogenic *Botrytis* species are likely to interact with cultivated plants, for example a foliar pathogen of *Hosta* (also in the order Asparagales) that is closely related to *B. tulipae* [30]. This new species, *B. deweyae*, represents the first novel and morphologically distinctive species to be identified in Europe for many years, as compared with recently described *B. pseudocinerea* that is genetically but not morphologically distinct from *B. cinerea* [31]. It is likely that *B. deweyae* has evaded detection as 1) the critical diagnostic morphological feature of macroconidia do not seem to develop on infected plant material nor are they produced from laboratory cultures except under a specific environmental regime, 2) isolation from infected material has not employed sufficiently stringent surface sterilization to prevent overgrowth from less systemic/pathogenic fungi, 3) the disease symptoms are relatively subtle, have a short temporal manifestation and can easily be attributed to another causative agent, and 4) it is probable that this species has a highly restricted host range.

Whilst there have been some reports of investigations of ‘spring sickness’ disease by specialist growers of *Hemerocallis* [15], [17], the causative agent had not been identified although other fungal species, such as the yeast-like *Aureobasidium microstictum* (daylily leaf streak) have been implicated [17]. The rigorous surface-sterilisation procedure we employed in isolations is likely to have been an important factor in finding this new species, as other opportunistic microbial species colonising damaged tissue more superficially would have been removed. In our studies, *B. deweyae* was repeatedly isolated from diseased material over a 4 year period. Whilst our pathogenicity assays using detached leaf material and plantlets *in vitro* under laboratory conditions cannot fully replicate mature plants growing in gardens in the colder months of the year, the rapid development of similar leaf tissue necrosis suggests this species has the capacity to be the major contributor to initiating symptom development. Other microbial species, such as *A. microstictum*, may opportunistically infect as secondary colonisers and aggravate disease development. The rapidity with which plantlet destruction took place was surprising but this is likely to have been enhanced by the absence of any other microbial flora in these cultures. Numerous microbial species have been shown to act antagonistically to *Botrytis* infections of plants (reviewed in [32]). For instance, infection of *Lilium* by *B. elliptica* can be antagonised by specific bacteria [33]. Our hypothesis that *B. deweyae* is the main agent of ‘spring sickness’ disease development is supported by a report of its closest relative, *B. elliptica*, infecting spring growth of *Hemerocallis fulva* in Korea [34]. In this case, symptoms were similar with necrosis and death of young leaf tissue, but it was accompanied by significant production of macroconidia. Tests *in vitro* indicated that *B. elliptica* was only capable of infecting *Hemerocalis* tissue after physical damage that wounded the plant [34]; this suggests that *B. elliptica* lacks the capacity to infect uninjured *Hemerocallis* tissues, as seen in our experimental controls. There have been no other reports of *B. elliptica* infecting *Hemerocallis*.

The infection of *Hemerocallis* with *B. elliptica* demonstrates that although this species has been most commonly associated with infections of bulbous true lily (Lilium, Liliaceae *sensu stricta*) - where it is a major disease of cultivated lily bulb crops [35] - it is also capable of infecting other distantly related petaloid monocots such as *Hemerocallis* (Hemerocallidaceae) and *Tricytris* (Convallariaceae) [34], [36]. There is also evidence that *B. elliptica* can infect dicotyledonous hosts [37], [38], albeit under unnatural conditions. Whether *B. deweyae* is an emergent pathogen that has evolved from a *B. elliptica* population that has undergone a host-shift to *Hemerocallis* is a matter that remains open for further study. The significant morphological and DNA sequence divergences from any described *B. elliptica* strain suggest that this would not have been a recent event. As 2 mating loci (*MAT1*) alleles are present in just 5 tested isolates, recent and rapid sympatric speciation from *B. elliptica* would appear unlikely. To date, only cultivated material from Britain has been examined but it is almost certain that *B. deweyae*, like its closest relative *B. elliptica* [35], [39], is cosmopolitan and will be detected in cultivated populations of hybrid *Hemerocallis* elsewhere in the world and perhaps also in native and naturalised *Hemerocallis* populations in nature.

Given the lack of any report of this species from indigenous *Hemerocallis* populations, its morphology and the nature of disease development, it is plausible that *B. deweyae* may be an endophyte that is undergoing the transition to a more aggressive pathogenic state. There was apparent variation in pathogenicity amongst three isolates tested on plantlets, with the isolate B1 being the most pathogenic, whilst isolates B2 and B4 were visibly less aggressive. Endophytic *Botrytis* have been identified using sequencing methods from naturalised populations of *Centaurea stoebe* [40], clearly demonstrating that outside of their native range plant species can retain or acquire endophytic *Botrytis* species. Five putative new endophytic species were discovered in this study alone. Intriguingly, there is also evidence that pathogenic *B. cinerea* (grey mould) can exist as a systemic endophyte without causing pathogenesis in plants [5], [6]. By some mechanism, *B. cinerea* must be capable of substantially down-regulating gene expression responsible for aggressive, necrotrophic pathogenesis thereby permitting it to co-exist and grow within plant tissues without damaging them or even triggering defence responses. In the case of *B. deweyae* the absence of identifiable macroconidia in nature, after growth on the host *in vitro* and even the absence of sporulation on plates (except under specific conditions) suggests that this development is usually highly suppressed, as might be expected of an endophytic lifestyle [4]. In common with other specialised *Botrytis* species that infect petaloid monocotyledons [35], additional studies are required to identify the precise details of the life cycle. Whether, as in *B. elliptica* [39], [41], sexual reproduction is important in natural populations of *B. deweyae* requires further investigations.

As this disease has become prevalent only in the past 20 years, one factor that may be precipitating the evolution of pathogenesis is the increasing loss of genetic diversity in the host plant through in-breeding of the cultivated hybrid lines, as demonstrated by an AFLP study [42]. It is known from studies of *Colletotrichum* that the ability for a fungal isolate to exhibit pathogenic or mutualistic behaviour can depend on the host genotype [43]. The emergence of in-breeding depression and loss of vigour amongst *Hemerocallis* hybrids may favour the emergence of strains that are able to effectively take advantage of weakened resistance systems in the plant and hence exploit the host in a more parasitic manner. In hybrid daylily, it is conceivable that the effects of combining divergent genomes followed by multiple generations of in-breeding and selection for novel traits have compromised the effectiveness of mechanisms that regulate pathogen resistance and endophyte activity *in planta*. Such a biotic environment may facilitate the emergence of new diseases, including transitions from endophytes to parasites and host shifts.

Other factors may have assisted the emergence of this disease. The clonal propagation of cultivars by division, which creates large wounds, and the tendency to plant *Hemerocallis* as monocultures or near-monocultures by enthusiasts may assist spread of strains with emerging pathogenic tendencies. A role for bulb mites (for example members of the genus *Rhizoglyphus*) in spreading fungal material such as mycelium fragments to new infection sites appears plausible [17], [44], [45]. Anecdotal reports suggest that both treatment by both acaricides and fungicides may reduce the incidence of ‘spring sickness’, supporting this view [17]. Young, emerging leaf tissues in late winter and spring may be particularly vulnerable to pathogenesis not only due to injury by fluctuating environments (such as tissue damage by freezing weather) but also due to rapid mobilisation of stored compounds from the tuberous roots to sustain new growth. Both factors may tip the balance in favour of fungal development.

Our discovery suggests that other potentially important pathogenic fungi could be easily overlooked and many are likely to remain unknown to science. The large reservoirs of endophytic fungi residing in plants [46], for example those found in wild grasses (Poaceae) [47], may be a source of important biological diversity for the evolution of new plant pathogens, especially with continued erosion of genetic diversity in cultivated plants and in fragmented natural habitats [1]. As threats from emerging pathogens to crop and wild plant resources continue to grow, investment in surveillance and detection systems for new plant diseases should be made a priority. This case in particular demonstrates that diagnostic tools using immunological and DNA sequencing methods, in combination with more conventional morphological and pathology assays, will need to work together in the future if other new plant diseases are to be identified effectively. Widespread ‘cryptic’ diseases of this kind with a small detrimental impact on the host may be important indicators of host and pathogen groups in which more virulent diseases are likely to be emergent.

## Materials and Methods

Permission to use plant material for the study was provided by the owner of the private collections. The studies did not involve endangered or protected species.

### Isolation from Plant Material

Samples were collected from collections of hybrid *Hemerocallis* growing in gardens in England, from Oxfordshire, Wiltshire and Somerset. Pieces of asymptomatic and symptomatic leaf tissue, each approximately 6 mm^2^, were surface-sterilized by immersing in 30% (v/v) sodium hypochlorite-based domestic bleach containing detergent (Parazone; Jeyes, Cambridge, UK) for 30 min followed by 4×5 min washes in sterile distilled water. Surface-sterilized leaf pieces were plated out on 2% (w/v) malt extract agar (MEA) (Oxoid, Basingstoke, UK) and grown under natural ambient light. The 6 isolates, labelled B1, B2, B4, B5, B6 and P1, were routinely grown on 2% MEA and stock cultures were maintained at 4°C in the dark. Stocks were also preserved as excised mycelial material from plate cultures in Eppendorfs in sterile 50% (v/v) glycerol at −80°C.

### Immunological Tests of Fungal Isolates and Plant Extracts for *Botrytis* Antigens

Plate cultures of fungal isolates were washed with 5 ml phosphate buffered saline, pH 7.4 (Sigma) plus 0.05% Tween 20 (Sigma) (PBST). 1 ml was removed by suction, centrifuged briefly at 13,000×*g* in a microcentrifuge and 400 µl of the supernatant tested with EnviroLogix Botrytis QuickStixs (Portland, Maine, USA) which employs the anti-*Botrytis*-monoclonal antibody, BC-12.CA4, raised and employed in previous studies [18], [19], [20]. Tests were performed by incubating a QuickStix in the supernatant for 10 minutes, the lower pad was then removed and the intensity of the test line (signal intensity, SI) was determined using an EnviroLogix Quickstix reader [19], [20]. Extracts of leaf tissues were made by crushing leaf material in an extraction bag (Noegen, UK) with PBST, 1∶5 (w/v). 400 µl of the resulting non-particulate extract was tested with EnviroLogix *Botrytis* QuickStix system as above.

### DNA Extraction, PCR Amplification and Sequencing

Genomic DNA was extracted from plugs of cultured mycelial material that were frozen and ground in liquid nitrogen. DNA was purified from tissue powder using the DNEasy kit (Qiagen, Manchester, UK) and quantified using a Nanodrop spectrophotometer. PCRs were performed using approximately 4–12 ng genomic DNA at in a 50 µl reaction using the proof-reading Phusion polymerase kit (Finnzymes, Thermo Scientific, UK) according to the manufacturer’s instructions. Cycling conditions for the amplifications were as follows:

98°C for 1 minute; 10 cycles of 98°C for 10 seconds, primer-specific annealing temperature 1 for 30 seconds, extension at 72°C for 45 seconds; 15 cycles of 98°C for 10 seconds, primer-specific annealing temperature 2 for 30 seconds, extension at 72°C for 45 seconds; final extension at 72°C for 5 minutes.

The 18S rRNA sequence was amplified using *ITS1* primers previously described [21] (Table S6 in File S1). The primer-specific annealing temperatures 1 and 2 were 60°C and 56°C respectively. The amplification of the *G3PDH, HSP60* and *RPB2* sequences was achieved using primers for the genus *Botrytis* described in [19] (Table S6 in File S1). Primer-specific annealing temperatures 1 and 2 were 62°C and 60°C respectively for *G3PDH* and *HSP60*, and 60°C and 56°C respectively for *RPB2*. *NEP1* was amplified using primers NEP1for and NEP1revB [22] (Table S6 in File S1) with primer-specific annealing temperatures 1 and 2 of 62°C and 60°C respectively. *NEP2* was amplified using primers NEP2forE and NEP2revE (Table S6 in File S1) [22] with primer-specific annealing temperatures 1 and 2 of 62°C and 60°C respectively. PCRs were run on 1.5% (w/v) agarose gels, the bands were cut out with a clean razor and extracted using Qiaex II kit (Qiagen, Manchester, UK). Purified PCR fragments were then cloned into pJET sequencing vector (CloneJET PCR cloning kit; Thermo Scientific, UK) and transformed into *E. coli* DH5α competent cells. Transformants were selected on LB-ampicillin plates (with 100 µg ampicillin per ml of medium) and colonies with inserts identified by PCR screening. Plasmid DNA was extracted from selected colonies using GeneJET Plasmid Miniprep Kit (Thermo Scientific, UK) and sequenced by Source Biosciences (Nottingham, UK).

Genomic DNA was extracted from leaf material showing symptoms and from leaf material of the same cultivar without symptoms, collected on the same day. Leaf material was derived from the cultivars ‘Gerda Brooker’ and ‘Free Bird’. Small sections of leaf material approximately 2×2 cm were ground to a fine powder in liquid nitrogen. DNA was purified from tissue powder using the DNEasy kit (Qiagen, Manchester, UK) and quantified using Nanodrop spectrophotometer. PCRs were performed using approximately 100 ng genomic DNA as template at in a 50 µl reaction using the proof-reading Phusion polymerase kit (Finnzymes, Thermo Scientific, UK) according to the manufacturer’s instructions. *NEP1* primers that amplify the promoter to 3′ end - NEP1(−207for) and NEP1(+1124rev) - [22] (Table S6 in File S1) were used in a primary PCR with cycling as follows.

98°C for 1 minute; 10 cycles of 98°C for 20 seconds, 64°C for 30 seconds, extension at 72°C for 45 seconds; 10 cycles of 98°C for 20 seconds, 60°C for 30 seconds, extension at 72°C for 45 seconds; 15 cycles of 98°C for 20 seconds, 58°C for 30 seconds, extension at 72°C for 45 seconds; final extension at 72°C for 5 minutes.

A 2 µl aliquot of primary PCR was then used as a template in a 50 µl reaction with NEP1for and NEP1revB primers [22] (Table S6 in File S1) as follows:

98°C for 1 minute; 15 cycles of 98°C for 10 seconds, 64°C for 30 seconds, extension at 72°C for 20 seconds; 15 cycles of 98°C for 10 seconds, 60°C for 30 seconds, extension at 72°C for 20 seconds; final extension at 72°C for 5 minutes.

PCRs were run on 1.5% (w/v) agarose gels, the PCR fragments were then removed, purified, cloned and sequenced as described above.

### Phylogenetic Analysis

We obtained sequences of *Botrytis* genes from GenBank (www.ncbi.nlm.nih.gov/genbank) and aligned them with newly sequenced genes from the six isolates using MUSCLE [48]. Phylogenies for single gene and combined datasets were reconstructed using a maximum-likelihood inference conducted with RAxML version 7.2.6 [49] via the raxmlGUI interface [50]. We conducted five independent runs from different starting points to assess convergence within two likelihood units of the best tree, which was consistently selected. The parameters of partition were allowed to vary independently under the GTRGAMMA model of evolution as implemented in RAxML. Maximum-likelihood nodal support was calculated by analysing 1000 bootstrap replicates.

### Induction of Sporulation and Sclerotia Formation

To determine whether presence of host material stimulated macroconidia production, 1% water agar on which surface-sterilized pieces of greenhouse-grown *Hemerocallis* ‘Jurassic Spider’ young leaf tissue had been placed. Plates were also made with 1% water agar with 0.5 ml plant extract added. The plant extract was made by thoroughly crushing young leaf tissue in an extraction bag (Neogen, Ayr, UK) with PBST at 1∶5 (w/v). The extract was passed through a 0.25 micron filter (Millipore) attached to a syringe for sterilisation before addition to molten agar. To attempt to stimulate production of macroconidia, MEA plates were incubated without light and under a mixture of day light and near UV light at room temperature (∼20°C) for 7 to 10 days. Plates were also incubated in the dark except for a continuous near-UV light source. Isolates were also grown on oatmeal agar (OMA) (Difco, Becton Dickinson BV, Breda, The Netherlands), potato dextrose agar (PDA) (Oxoid, Basingstoke, UK), Czapeks Dox medium (Oxoid, Basingstoke, UK), V8 agar (prepared with 200 ml V8 vegetable juice (Campbell Soup Company, Camden, NJ, USA), 20 g agar, 800 ml water, pH adjusted to 6.0 with NaOH).

### Light Microscopy

Sporulating structures were mounted on slides with filter-sterilised MilliQ water. Observations were made with a Nikon Eclipse 90i (Nikon Instruments, Badhoevedorp, The Netherlands) compound microscope with a Nikon DS-5MC camera attached. Measurements were performed using N.I.S. Elements AR 2.30 software (Nikon Instruments, Badhoevedorp, The Netherlands).

### Electron Microscopy

Spores were removed from the edges of mature cultures of B2 and B4 using a sticky pad mounted in a SEM stub. Samples were directly coated with gold/palladium in a Polaron SC7640 sputter coating unit (Quorum Technologies, Ashford, UK). Spores were also trapped using poly-lysine coated slides and subsequently treated in osmium tetroxide vapour for 3 hours, followed by 4% paraformaldehyde in phosphate buffer for 3 hours, dehydrated then subsequently sputter coating as described. Images were taken using a JEOL JSM-5510 scanning electron microscope unit (JEOL, Welwyn Garden City, UK) operating at 15 kV.

### Nomenclature

The electronic version of this article in Portable Document Format (PDF) in a work with an ISSN or ISBN will represent a published work according to the International Code of Nomenclature for algae, fungi, and plants, and hence the new names contained in the electronic publication of a PLOS ONE article are effectively published under that Code from the electronic edition alone, so there is no longer any need to provide printed copies.

In addition, new names contained in this work have been submitted to MycoBank from where they will be made available to the Global Names Index. The unique MycoBank number can be resolved and the associated information viewed through any standard web browser by appending the MycoBank number contained in this publication to the prefix http://www.mycobank.org/MB/. The online version of this work is archived and available from the following digital repositories: PubMed Central and LOCKSS etc.

### Pathogenicity Assays

Pathogenicity tests were carried out using detached leaves of the following plants: *Alstroemeria* hybrid; *Tricyrtis formosana*; *Lilium* Oriental hybrid; *Hemerocallis fulva* and *Hemerocallis* ‘Jurassic Spider’. Leaves were placed on 0.3% water agar in large Petri dishes Four replicates were made with the leaf adaxial and abaxial sides upwards. The exposed surfaces were inoculated with a 0.5 cm mycelial plug from MEA cultures of isolates B1, B2 and B4. The Petri dishes were sealed and incubated at 25°C under 12 hours light and monitored for up to 14 days. As a control, plugs of *B. elliptica* (isolate 9601), grown on MEA agar under identical conditions, were used. A sterile MEA agar control was also performed.

Macroconidia of B4 and B5 from sporulating plates were collected in sterile potato dextrose broth (1.2 g/l) and adjusted to concentrations of 1×10^5^ and 1×10^6^ ml^−1^. Excised *Hemerocallis* leaves on water agar were inoculated with 5 µl droplets of spore suspensions on their upper sides and three replicates performed for each. The material was sealed in a plastic box with a >90% humidity at 25°C and monitored for up to 10 days after inoculation.

Pathogenicity tests were also carried out on axenic plantlets of *Hemerocallis* ‘Jurassic Spider’ as follows. Previously established cultures of *Hemerocallis* ‘Jurassic Spider’ were maintained at 4°C on a 0.6% agarose growth medium composed of Murashige and Skoog macroelements [51] with Heller’s microelements [52] and 5 ml/litre of 1% ferric ammonium citrate solution, adjusted to pH 5.5 before autoclaving. The clumps of shoots were divided, with the leaves and roots trimmed. The plantlets derived from division were grown in Magenta boxes containing sterilised 100 ml vermiculite plus 80 ml liquid growth medium (as above, but lacking the 0.6% agarose). In each box, 5 divisions were planted and chilled at 4°C in the dark for 2 weeks, then removed in a well-lit growth room at 20°C with 16 hour photoperiod for 2 weeks to allow wound healing and establishment prior to inoculation. Individual plantlets were inoculated with a 0.5 mm mycelial plug from a plate culture of one of the fungal isolates (B1, B2, B4) and incubated at 25°C under 16 hours direct light in a growth room for up to 10 days. For controls, *B. elliptica* (isolate 9601) and sterile MEA plugs were used. For each fungal isolate and for each control, three replicate Magenta boxes were inoculated.

To confirm *Botrytis* infection of *Hemerocallis* tissue, extracts of plant tissues were made by crushing sections of aerial material in an extraction bag (Neogen, UK) with PBST, 1∶5 (w/v). 400 µl of the resulting non-particulate extract was tested with EnviroLogix *Botrytis* QuickStix system as above.

To determine if pathogenicity varied depending on the host cultivar that was challenged, plantlets of a range of cultivars were tested using the same method except that one plantlet per Magenta box was used. Two replicates were performed for each plant genotype. These pathogenicity assays were all performed with the B1 isolate. The 15 cultivars were as follows: ‘Running Late’, ‘Lavender Curls’, ‘Dark Mosaic’, ‘Heavenly Flight of Angels’, ‘Golden Chimes’, ‘Barbara’, ‘Party Array’, ‘Rococo’, ‘Bo Knows’, ‘Cayenne’, ‘Corky’, ‘Miss Jessie’, ‘flava clone 3’, ‘Persian Pattern’, ‘Jellyfish Jealousy’.

### Identification of Mating Types and Sexual Crosses

Five isolates were analysed (B1, B2, B4, B5 and P1) to identify the mating type alleles. Gentra Puregene DNA purification kit (Qiagen, Venlo, The Netherlands) was used for DNA extraction from freeze-dried mycelia following the manufacturer’s instructions. 10*–*50 ng genomic DNA was used in 25 µl reaction volume. PCRs were performed with GoTaq polymerase (Promega) according to manufacturer’s instructions. Primers used were *MAT1-1* forward/reverse and *MAT1-2* forward/reverse (Table S6 in File S1). Amplification conditions were as follows: 95°C 5 minutes, then 35 cycles of 94°C for 30 seconds, 52°C for 30 seconds and 72°C for 2 minutes, followed with a final extension of 72°C for 5 minutes. PCR products were visualised on gel to determine the mating type of each isolate. Crosses were set up between isolates carrying different mating types [24]. Isolates B1 and B5 (each of the *MAT1-1* mating type) were mated with isolates B2 and B4 (each of the *MAT1-2* mating type). To develop sclerotia, strains were plated on oatmeal agar and incubated in darkness for 1 month at 15°C, followed by incubation at 0°C in darkness for 1 month. For mating, sclerotia were sampled from the plates, rinsed in water with a soft toothbrush and place in a 6-well microtitre plate. Mycelial cultures were flooded with sterile water and a suspension of mycelial fragments and microconidia was obtained by gently rubbing the surface with a spatula. The sclerotia were fertilised by addition of this suspension at 3 ml per well. Reciprocal crosses were set up in this manner, with each partner as a female (sclerotial) or male (microconidial) parent. As a control, sclerotia that were not exposed to microconidia and kept in sterile water were used. The microtitre plates were sealed and incubated at 12°C in normal artificial light with a 12 hour photoperiod.

## Supporting Information

Figure S1 **Exceptional examples of spring foliage of** ***Hemerocallis*** **that is exhibiting symptoms of ‘spring sickness’ and also extensive, visible fungal growth.** A. Immature emergent foliage of a *Hemerocallis* cultivar (*H.* ‘Ruby Storm’), showing severe necrosis and chlorosis. *Botrytis deweyae* was isolated from this material. Scale bar indicates 1 cm. B. Close-up of fungal growth of *B. deweyae* on infected *Hemerocallis* (*H.* ‘Gerda Brooker’) leaf material. The fungal growth is showing production of microconidia. Scale bar indicates 500 microns.
(TIF)

Figure S2 **Phylogeny of** ***Botrytis*** **using** ***NEP1*** **sequences.** The phylogenetic position of *B. deweyae* - B1 (type) isolate - is underlined. The phylogeny was generated using *Sclerotinia sclerotiorum* as the outgroup.
(TIFF)

Figure S3 **Phylogeny of** ***Botrytis*** **using** ***NEP2*** **sequences.** The phylogenetic position of *B. deweyae* - B1 (type) isolate - is underlined. The phylogeny was generated using *Sclerotinia sclerotiorum* as the outgroup.
(TIFF)

Figure S4 **Phylogeny of** ***Botrytis*** **using** ***G3PDH*** **sequences.** The phylogenetic position of *B. deweyae* - B1 (type) isolate - is underlined. The phylogeny was generated using the *Sclerotinia* fungal group as the outgroup.
(TIFF)

Figure S5 **Phylogeny of** ***Botrytis*** **using** ***HSP60*** **sequences.** The phylogenetic position of *B. deweyae* - B1 (type) isolate - is underlined. The phylogeny was generated using the *Sclerotinia* fungal group as the outgroup.
(TIFF)

Figure S6 **Phylogeny of** ***Botrytis*** **using** ***RPB2*** **sequences.** The phylogenetic position of *B. deweyae* - B1 (type) isolate - is underlined. The phylogeny was generated using the *Sclerotinia* fungal group as the outgroup.
(TIFF)

Figure S7 **Phylogeny of** ***Botrytis NEP1*** **sequences amplified from infections of** ***Botrytis deweyae in planta*** **.** The plant material was showing ‘spring sickness’ symptoms. Phylogenetic positions of sequences of *NEP1* from *B. deweyae* from two different cultivars showing ‘spring sickness’ are shown in red.
(TIFF)

Figure S8 **Scanning electron micrograph of a macroconidia of** ***Botrytis deweyae***. Scale bar indicates 2 µm.
(TIF)

File S1 **Supporting information file containing Tables S1–S6.**
(DOCX)
