# Supplementary figures and images for: A Novel Botrytis Species Is Associated with a Newly Emergent Foliar Disease in Cultivated Hemerocallis
Source: PLoS One. 2014 Jun 2;9(6):e89272. doi: 10.1371/journal.pone.0089272 (PMC4041564; doi:10.1371/journal.pone.0089272)

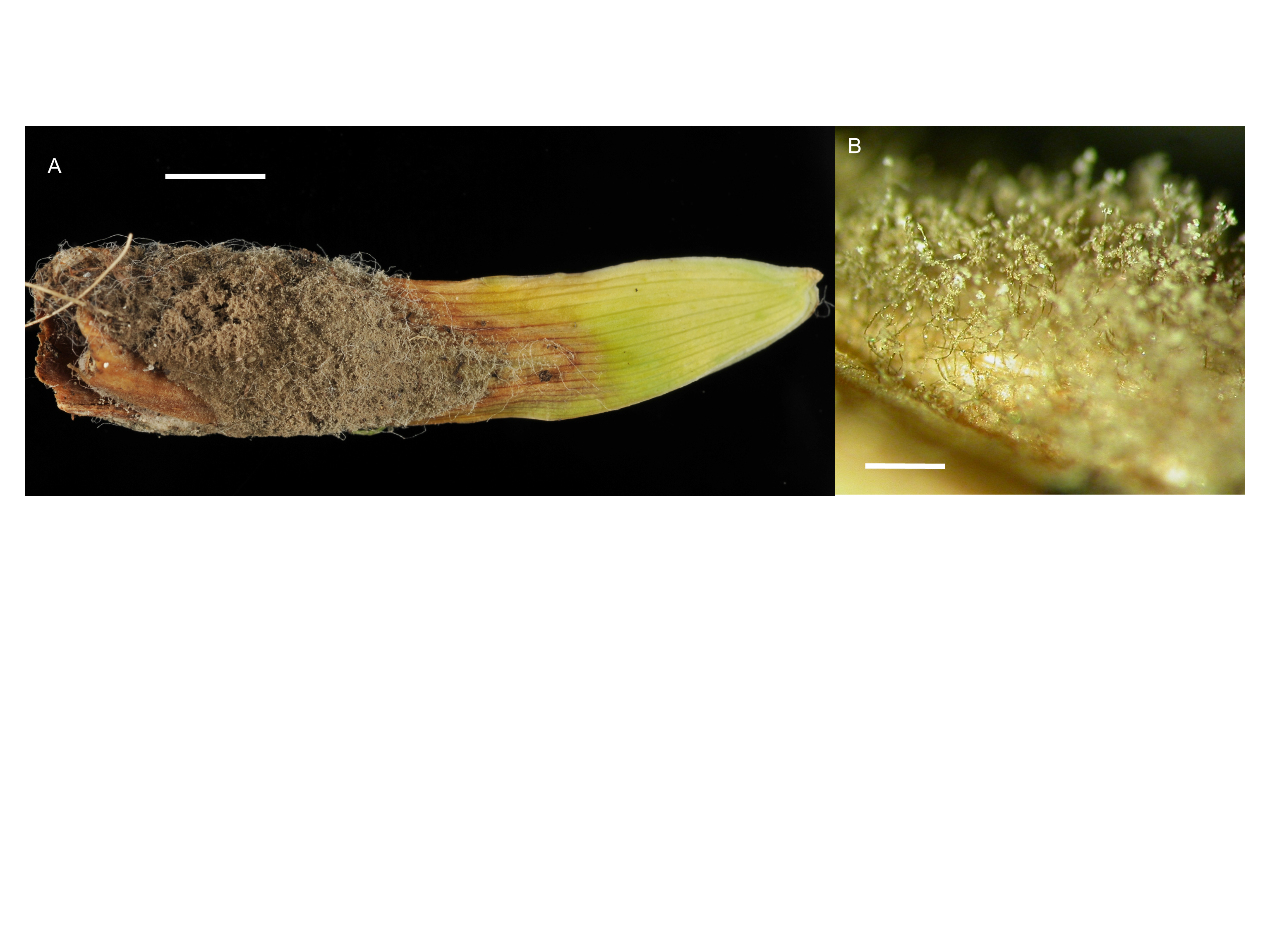

Supplement: Figure S1 — Exceptional examples of spring foliage of Hemerocallis that is exhibiting symptoms of ‘spring sickness’ and also extensive, visible fungal growth. A. Immature emergent foliage of a Hemerocallis cultivar (H. ‘Ruby Storm’), showing severe necrosis and chlorosis. Botrytis deweyae was isolated from this material. Scale bar indicates 1 cm. B. Close-up of fungal growth of B. deweyae on infected Hemerocallis (H. ‘Gerda Brooker’) leaf material. The fungal growth is showing production of microconidia. Scale bar indicates 500 microns. (TIF) [file pone.0089272.s001.tif]

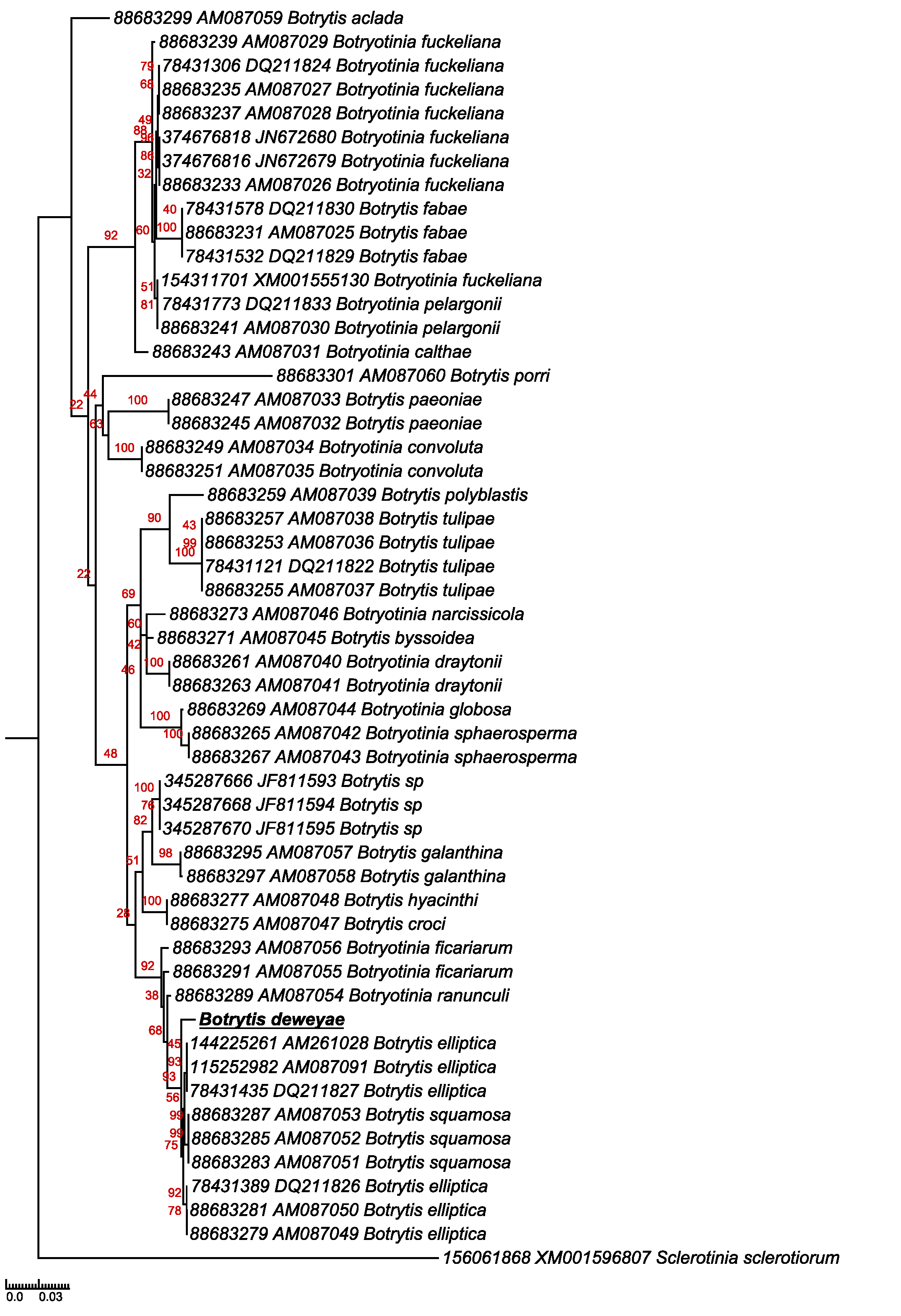

Supplement: Figure S2 — Phylogeny of Botrytis using NEP1 sequences. The phylogenetic position of B. deweyae - B1 (type) isolate - is underlined. The phylogeny was generated using Sclerotinia sclerotiorum as the outgroup. (TIFF) [file pone.0089272.s002.tiff]

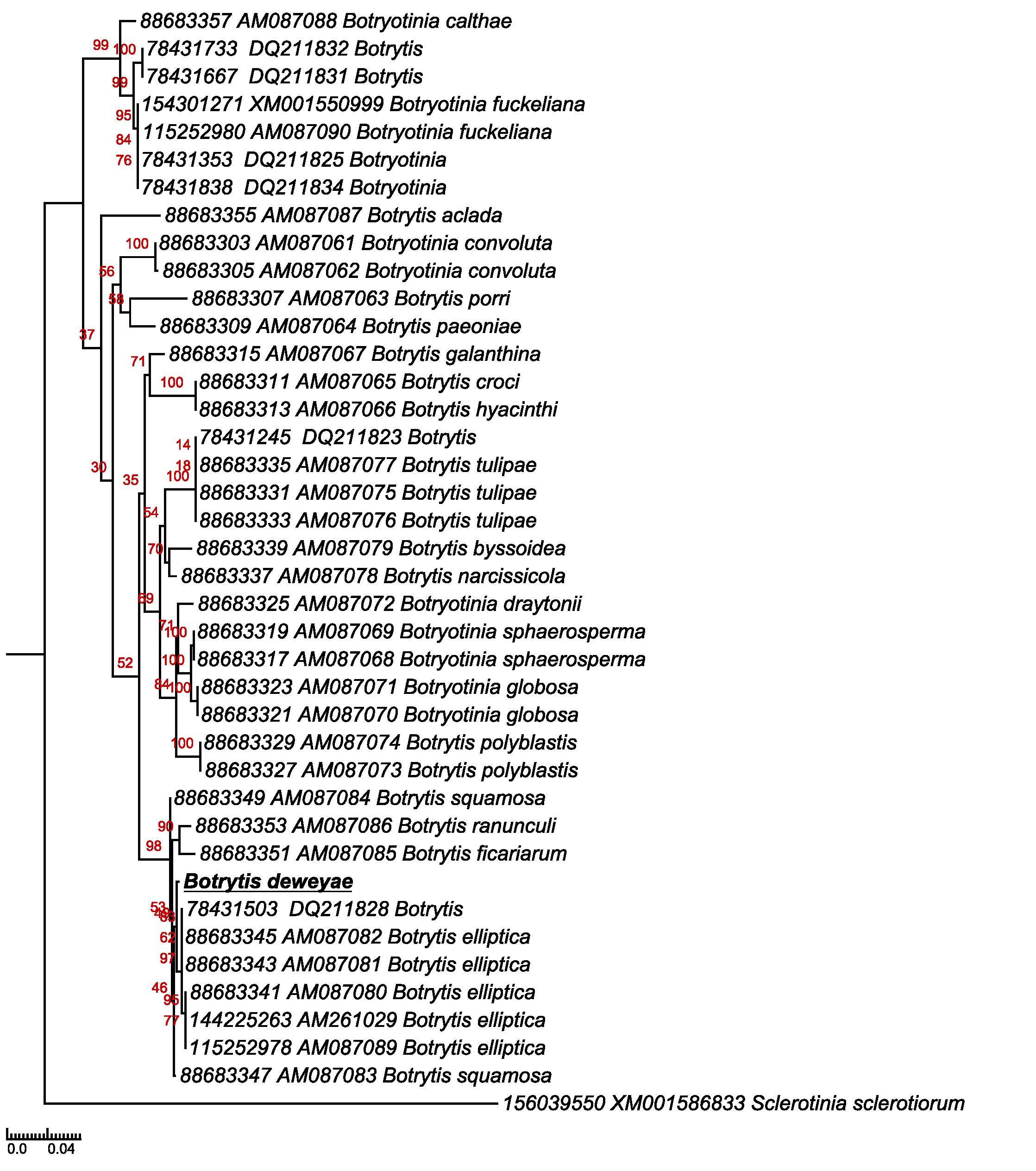

Supplement: Figure S3 — Phylogeny of Botrytis using NEP2 sequences. The phylogenetic position of B. deweyae - B1 (type) isolate - is underlined. The phylogeny was generated using Sclerotinia sclerotiorum as the outgroup. (TIFF) [file pone.0089272.s003.tiff]

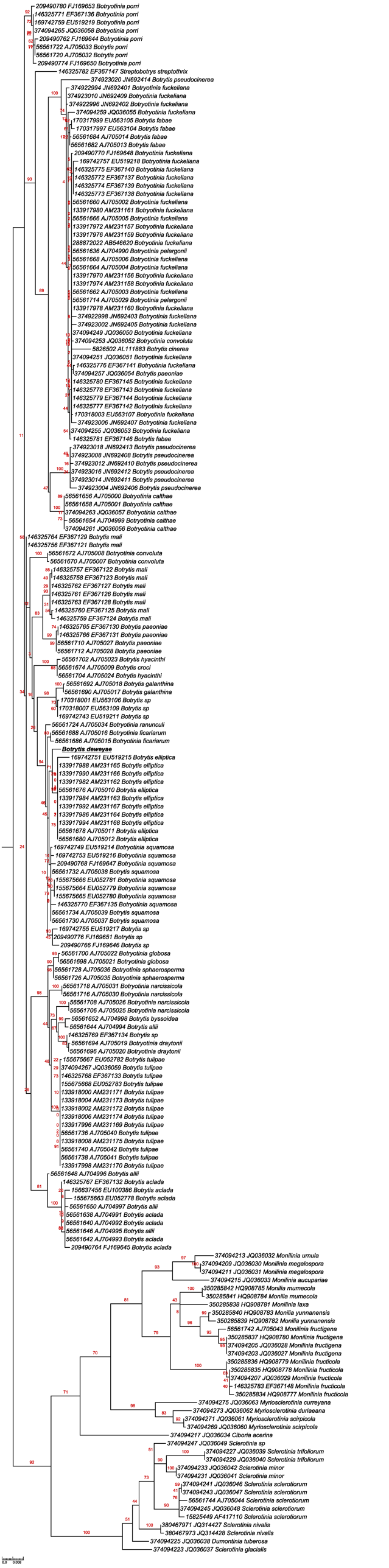

Supplement: Figure S4 — Phylogeny of Botrytis using G3PDH sequences. The phylogenetic position of B. deweyae - B1 (type) isolate - is underlined. The phylogeny was generated using the Sclerotinia fungal group as the outgroup. (TIFF) [file pone.0089272.s004.tiff]

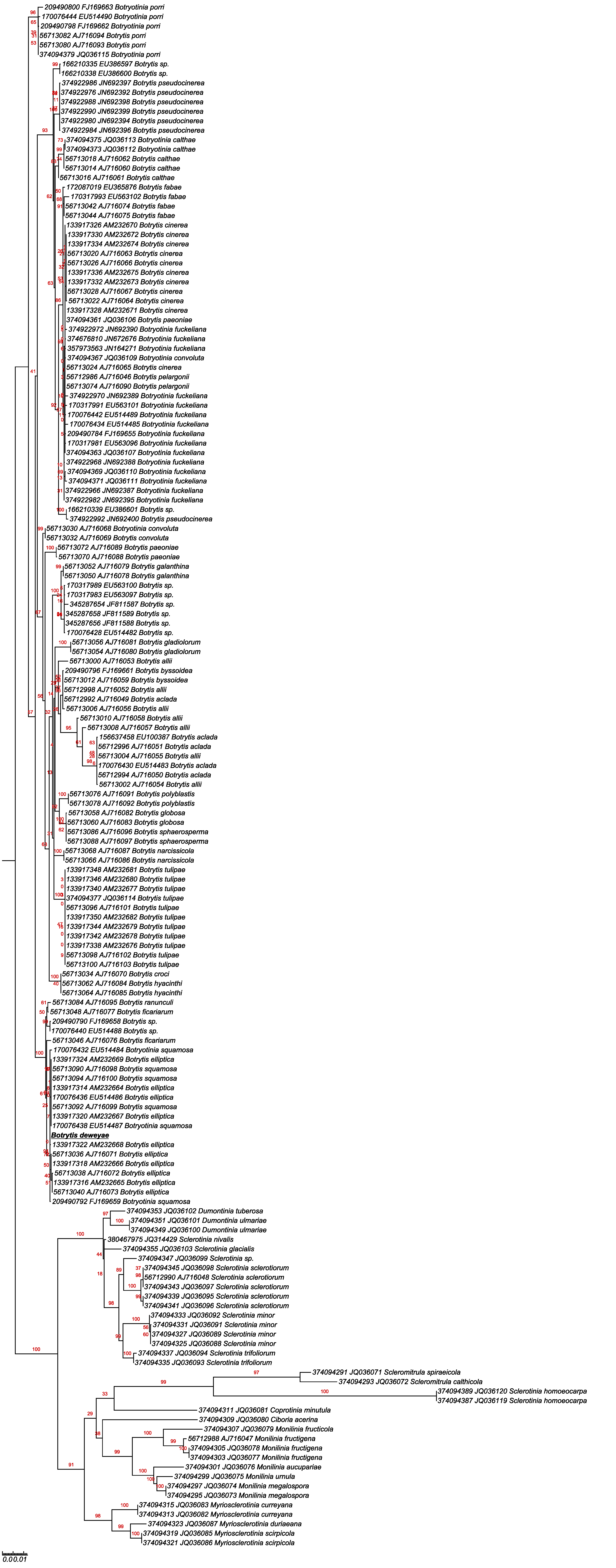

Supplement: Figure S5 — Phylogeny of Botrytis using HSP60 sequences. The phylogenetic position of B. deweyae - B1 (type) isolate - is underlined. The phylogeny was generated using the Sclerotinia fungal group as the outgroup. (TIFF) [file pone.0089272.s005.tiff]

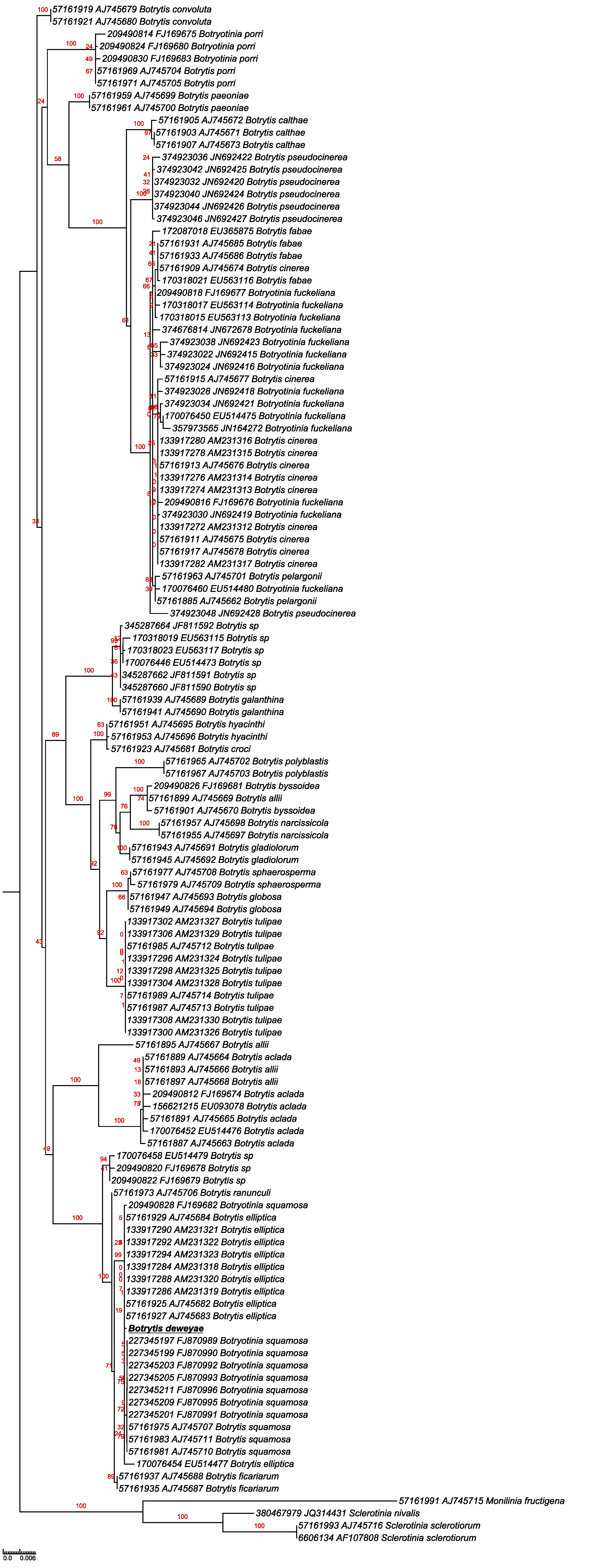

Supplement: Figure S6 — Phylogeny of Botrytis using RPB2 sequences. The phylogenetic position of B. deweyae - B1 (type) isolate - is underlined. The phylogeny was generated using the Sclerotinia fungal group as the outgroup. (TIFF) [file pone.0089272.s006.tiff]

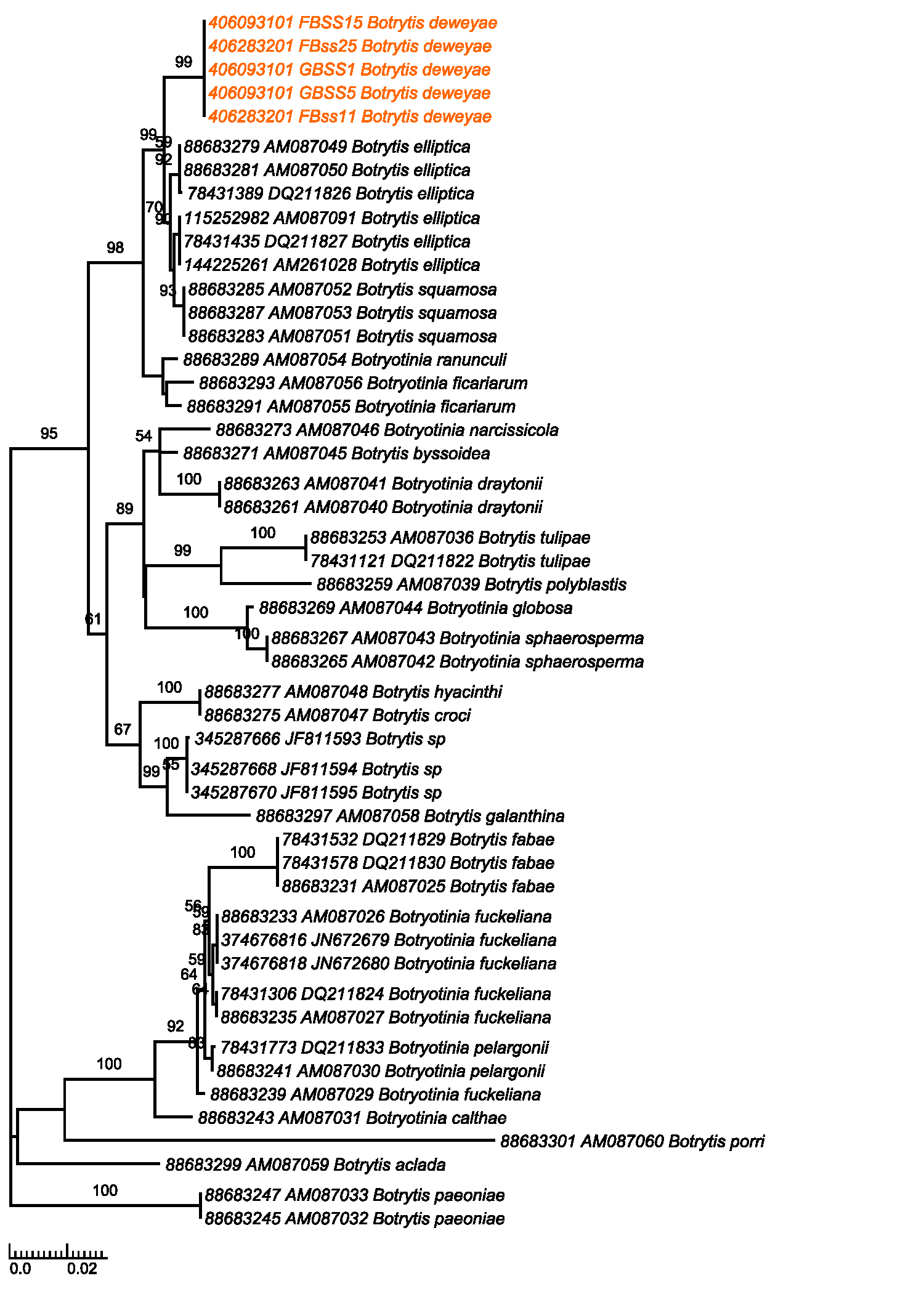

Supplement: Figure S7 — Phylogeny of Botrytis NEP1 sequences amplified from infections of Botrytis deweyae in planta . The plant material was showing ‘spring sickness’ symptoms. Phylogenetic positions of sequences of NEP1 from B. deweyae from two different cultivars showing ‘spring sickness’ are shown in red. (TIFF) [file pone.0089272.s007.tiff]
