# Supplementary material for: A Novel Botrytis Species Is Associated with a Newly Emergent Foliar Disease in Cultivated Hemerocallis
Source: PLoS One. 2014 Jun 2;9(6):e89272. doi: 10.1371/journal.pone.0089272 (PMC4041564; doi:10.1371/journal.pone.0089272)
Supplement: File S1 — Supporting information file containing Tables S1–S6. (DOCX) [file pone.0089272.s009.docx]

**Supporting information text and tables for Grant-Downton *et al.***

**Figure S1.** Exceptional examples of spring foliage of *Hemerocallis* that is exhibiting symptoms of ‘spring sickness’ and also extensive, visible fungal growth. A. Immature emergent foliage of a *Hemerocallis* cultivar (*H.* ‘Ruby Storm’), showing severe necrosis and chlorosis. *Botrytis deweyae* was isolated from this material. Scale bar indicates 1 cm. B**.** Close-up of fungal growth of *B. deweyae* on infected *Hemerocallis* (*H.* ‘Gerda Brooker’) leaf material. The fungal growth is showing production of microconidia. Scale bar indicates 500 microns.

**Figure S2. Phylogeny of *Botrytis* using *NEP1* sequences.** The phylogenetic position of *B. deweyae* -B1 (type) isolate - is underlined. The phylogeny was generated using *Sclerotinia sclerotiorum* as the outgroup.

**Figure S3. Phylogeny of *Botrytis* using *NEP2* sequences.** The phylogenetic position of *B. deweyae* - B1 (type) isolate - is underlined. The phylogeny was generated using *Sclerotinia sclerotiorum* as the outgroup.

**Figure S4. Phylogeny of *Botrytis* using *G3PDH* sequences.** The phylogenetic position of *B. deweyae* - B1 (type) isolate - is underlined. The phylogeny was generated using the *Sclerotinia* fungal group as the outgroup.

**Figure S5.** **Phylogeny of *Botrytis* using *HSP60* sequences.** The phylogenetic position of *B. deweyae* - B1 (type) isolate - is underlined. The phylogeny was generated using the *Sclerotinia* fungal group as the outgroup.

**Figure S6.** **Phylogeny of *Botrytis* using *RPB2* sequences.** The phylogenetic position of *B. deweyae* - B1 (type) isolate - is underlined. The phylogeny was generated using the *Sclerotinia* fungal group as the outgroup.

**Figure S7. Phylogeny of *Botrytis* *NEP1* sequences amplified from infections of *Botrytis deweyae* *in planta*.** The plant material was showing ‘spring sickness’ symptoms. Phylogenetic positions of sequences of *NEP1* from *B. deweyae* from two different cultivars showing ‘spring sickness’ are shown in red.

**Figure S8. Scanning electron micrograph of a macroconidia of *Botrytis deweyae.*** Scale bar indicates 2 μm.

**Table S1. Formation of sclerotia and sclerotia-like structures from different *Botrytis deweyae* isolates.** The isolates were grown on different media and sclerotia development noted after 27 days at 15°C in darkness.

| **Medium** | **B1 isolate** | **B2 isolate** | **B4 isolate** | **B5 isolate** |
| --- | --- | --- | --- | --- |
| Oatmeal agar | Yes | Yes | No | Yes |
| Czapek Dox | Yes | Yes | No | No |
| V8 juice agar | Yes | Yes | No | No |
| MEA | No | Yes | No | Yes |
| PDA | Yes | Yes | No | No |

**Table S2. Production of macroconidia (sporulation) from isolates of *Botrytis deweyae* grown on different media.** Colonies were grown at 20°C in darkness except for a UV light source, and examined for sporulation at 6 days and 12 days.

| **Medium** | **Days of exposure to UV in darkness** | **B1 isolate** | **B2 isolate** | **B4 isolate** | **B5 isolate** |
| --- | --- | --- | --- | --- | --- |
| Oatmeal agar | 6 | No sporulation | No sporulation | Sporulation | No sporulation |
| Czapek Dox | 6 | No sporulation | No sporulation | Sporulation | No sporulation |
| V8 juice agar | 6 | No sporulation | No sporulation | Sporulation | No sporulation |
| Oatmeal agar | 12 | No sporulation | No sporulation | Sporulation | Sporulation |
| Czapek Dox | 12 | No sporulation | No sporulation | Sporulation | No sporulation |
| V8 juice agar | 12 | Sporulation | No sporulation | Sporulation | No sporulation |

**Table S3.** Comparison of conidiation morphology of *Botrytis deweyae* to several other described species in the genus of major importance as widespread diseases of cultivated plants

| **Species** | **Conidiophores** | | | **Macroconidia** | | | |  |
| --- | --- | --- | --- | --- | --- | --- | --- | --- |
|  | **Colour** | **Length µm** | **Width µm** | **Colour** | **Shape** | **Surface** | **Length µm** | **Width µm** |
| ***Botrytis deweyae*** | medium brown | 3 – 4 | 10 – 20 | hyaline to medium brown | ellipsoid to ovoid, becoming oblong and 1-septate with age, or irregular and somewhat distorted | smooth | 6.5 - 18 | 3.5 – 11 |
| ***Botrytis cinerea*** Ellis (1971) | brown to pale brown | >2 | 16 – 30 | pale brown | ellipsoidal or obovoid | smooth | 6.0 – 18.0 | 4.0 – 11.0 |
| ***Botrytis cinerea*** Zhang *et al.* (2010) | n/d | n/d | n/d | brown | elliptical to ovoid | smooth | 7.0 – 14.0 | 6.0 – 13.0 |
| ***Botrytis cinerea*** Mirzaei *et al.* (2008) | n/d | n/d | n/d | n/d | n/d | n/d | 4.0 -20.0 | 2.0 -12.0 |
| ***Botrytis tulipae*** Ellis (1971) | n/d | n/d | n/d | n/d | n/d | n/d | 12.0 - 22.0 | 8.0 – 15.0 |
| ***Botrytis tulipae*** Sung *et al.* (2002) | pale brown | 0.7 – 1.0 | 14.0 – 20.0 | pale brown | ellipsoidal or obovoid | smooth | 13.8 -22.5 | 8.0 -12.5 |
| ***Botrytis elliptica*** Ellis (1971) | n/d | n/d | n/d | n/d | n/d | n/d | 16.0 -35.0 | 10.0 – 24.0 |
| ***Botrytis elliptica*** Chang *et al*. (2002) | n/d | n/d | n/d | hyaline to pale brown | ellipsoidal to obovate | n/d | 21.0 – 31.0 | 12.0 – 23.0 |

n/d – no data.

*Supplemental references for Table S3:*

Chang SW, Kim SK, Hwang BK (2001) Gray mould of daylily (*Hemerocallis fulva* L.) caused by *Botrytis elliptica* in Korea. *Plant Pathology Journal* 17: 305-307

Ellis MB (1971) Dematiaceous hyphomycetes. Commonw. Mycol. Inst., Kew, Surrey, England. 608 p.

Mirzaei S, Mohammadi Goltapeh E, Shams-Bakhsh M, Safaie N (2008) Identification of *Botrytis* spp. on plants grown in Iran. *Journal of Phytopathology* 156: 21-28

Zhang J, Zhang L, Li G-Q, Yang L, Jiang, D-H, Zhuang W-Y, Huang H-C (2010) *Botrytis sinoallii* – a new species of the grey mould pathogen on *Allium* crops in China. *Mycoscience* 51: 421-431

Sung KH, Wan GK, Weon DC, Hong GK (2002) Occurrence of tulip fire caused by *Botrytis* *tulipae* in Korea. *Plant Pathology Journal* 18: 106-108

**Table S4.** Results of inoculation of mycelial plugs of *B. deweyae* and *B. elliptica* on epidermal surface of leaf material of various monocotyledons.

| **Leaf material** | *B. deweyae* | *B. elliptica* | control (agar plug only) |
| --- | --- | --- | --- |
| *Hemerocallis* ‘Jurassic Spider’ and *Hemerocallis fulva* | Spreading lesions, often water-soaked | No lesions | No lesions |
| *Tricyrtis formosana* | No lesions | No lesions | No lesions |
| *Lilium* Oriental Hybrid | Slight water-soaked lesion only with B1 isolate, otherwise no lesion | Rapidly spreading water-soaked lesions | No lesions |
| *Alstroemeria* hybrid | No lesions | No lesions | No lesions |

**Table S5.** Results of PCR assays for identity of MAT1 alleles in different *Botrytis deweyae* isolates.

| **Isolate** | B1 isolate | B2 isolate | B4 isolate | B5 isolate | P1 isolate |
| --- | --- | --- | --- | --- | --- |
| **MAT locus** | *MAT1-1* | *MAT1-2* | *MAT1-2* | *MAT1-1* | *MAT1-2* |

**Table S6.** List of primers employed in PCRs in this study.

| **Primer name (for = forward primer, rev = reverse primer)** | **Primer sequence (5’ to 3’)** |
| --- | --- |
| G3PDH for | ATTGACATCGTCGCTGTCAACGA |
| G3PDH rev | ACCCCACTCGTTGTCGTACCA |
| HSP60 for | CAACAATTGAGATTTGCCCACAAG |
| HSP60 rev | GATGGATCCAGTGGTACCGAGCAT |
| ITS for | TCCGTAGGTGAACCTGCGG |
| ITS rev | TCCTCCGCTTATTGATATGC |
| RPB2 for | GATGATCGTGATCATTTCGG |
| RPB2 rev | CCCATAGCTTGCTTACCCAT |
| NEP1(−207)for | CACCTTGTGGGAGATTGTATGGGTGGATATACATC |
| NEP1(+1124)rev | GGTCACCTAATTTTGGCTTTCAGGGTC |
| NEP1for | CCAACGCAAAATTCCTTTCTATCC |
| NEP1revB | GTTGGCGAAGTTGTGGTCATTGAA |
| NEP2forE | TCATCATGGTTGCCTTCTCAAGAT |
| NEP2revE | AAGTAGCAGCTGCAAGATTGTTTG |
| MAT 1-1 F | CCAGCAGTAAATGCAGAAGAGCCAA |
| MAT 1-1 R | CATCATACCAGTGGACCAAGGAGG |
| MAT 1-2 F | GACTAGGAAAATGGGTACCGCATC |
| MAT 1-2 R | GAATGTGTAGAGATCCTGTTGTTG |
